# CD1d-Invariant Natural Killer T Cell-Based Cancer Immunotherapy: α-Galactosylceramide and Beyond

**DOI:** 10.3389/fimmu.2018.01519

**Published:** 2018-07-02

**Authors:** Lisa A. King, Roeland Lameris, Tanja D. de Gruijl, Hans J. van der Vliet

**Affiliations:** ^1^Department of Medical Oncology, VU University Medical Center and Cancer Center Amsterdam, Amsterdam, Netherlands; ^2^Lava Therapeutics, Den Bosch, Netherlands

**Keywords:** iNKT cells, CD1d, α-GalCer, glycolipids, cancer immunotherapy

## Abstract

CD1d-restricted invariant natural killer T (iNKT) cells are considered an attractive target for cancer immunotherapy. Upon their activation by glycolipid antigen and/or cytokines, iNKT cells can induce direct lysis of tumor cells but can also induce an antitumor immune response *via* their rapid production of proinflammatory cytokines that trigger the cytotoxic machinery of other components of the innate and adaptive immune system. Here, we provide an overview of various therapeutic approaches that have been evaluated or that are currently being developed and/or explored. These include administration of α-GalCer or alternative (glyco) lipid antigens, glycolipid-loaded antigen-presenting cells and liposomes, strategies that enhance CD1d expression levels or are based on ligation of CD1d, adoptive transfer of iNKT cells or chimeric antigen receptor iNKT cells, and tumor targeting of iNKT cells.

## The Invariant Natural Killer T (iNKT) Cell as Target for Cancer Immunotherapeutic Approaches

Invariant natural killer T cells belong to a population of T lymphocytes, which harbor distinct characteristics of both natural killer (NK) and T cells (1). These cells express a semi-invariant T cell receptor (TCR), in humans consisting of a Vα24-Jα18 chain paired with Vβ11, and NK cell markers (e.g., CD161 and NKG2D) (2). Two CD1d-restricted NKT cell subtypes exist, the classical (type I) iNKT and non-classical (type II) NKT subsets expressing a diverse TCR repertoire (1). Both subsets are able to secrete immunoregulatory cytokines upon glycolipid recognition presented *via* the human leukocyte antigen class I-related molecule CD1d (2). iNKT cells release, upon their interaction with CD1d, a broad spectrum of cytokines, which in turn activate T cells, NK cells, B cells, and dendritic cells (DCs), thereby initiating a T helper (Th) 1, Th2 or Th17 response (1, 3–6). The role that the type I CD1d-restricted iNKT cell population can play in the antitumor immune response will be the main focus of this review.

CD1d-restricted iNKT cells can play a role in mediating antitumor immunity in various ways: indirectly *via* recognition of glycolipid-loaded CD1d molecules expressed by antigen-presenting cells (APCs), directly *via* recognition of glycolipid loaded CD1d expressed by tumor cells and alternatively *via* a TCR-independent manner through cytokines (6, 7). In case of recognition of glycolipid-loaded CD1d on APCs, the antitumor effect is mediated *via* secretion of inflammatory cytokines. Ligation of glycolipid-loaded CD1d molecules by iNKT cells amplifies IL-12 production and, like CD4^+^ T helper cells, can induce maturation of DCs, conversely resulting in enhanced IFN-γ production by the interacting iNKT cells (8, 9). Secretion of these inflammatory cytokines in turn promotes the cytolytic function of cytotoxic CD8^+^ T cells and NK cells. In case of recognition of tumor cells expressing CD1d, iNKT cells can exert a direct antitumor effect *via* secretion of perforin and granzymes and death inducing receptors (e.g., Fas and TRAIL) reviewed by Bassiri et al. (9). Because of the cytotoxic capacity of iNKT cells and their ability to orchestrate pro- and anti-inflammatory immune responses, these cells are very attractive targets to exploit for cancer immunotherapy. Here, we will outline multiple strategies that can be used in order to promote iNKT cell based cancer immunotherapy.

## α-Galactosylceramide

Several glycolipids have been demonstrated to act as activating agents for both murine and human iNKT cells, of which, α-galactosylceramide (α-GalCer) is the best known and most intensely studied. This glycosphingolipid was originally isolated from the marine sponge *Agelas mauritianus* and activates iNKT cells in a very potent way (10). Upon activation with α-GalCer, iNKT cells secrete Th1, Th2, and Th17 cytokines, modulating immune responses against tumors, microbial infections, viral infections, and auto-immune diseases (3, 5, 11, 12).

α-GalCer-induced antitumor immune responses in several *in vivo* models using different tumor types (13). Subsequent clinical studies with α-GalCer did not show any adverse events but also did not result in clinically relevant antitumor effects in advanced cancer patients (14). The effect of α-GalCer may be limited by the relatively short-lived and in part antagonizing nature of the mix of Th1 and Th2 cytokines that is produced by activated iNKT cells, followed by long-term anergy of iNKT cells (10, 15, 16).

Changing the route of administration of α-GalCer may enhance efficacy. Direct intravenous administration of α-GalCer in patients with solid tumors led to an increase in serum cytokine levels but also to the disappearance of iNKT cells from the circulation within 24 h (14). Furthermore, upon repeated systemic administration of α-GalCer, increases in serum cytokine levels were no longer observed, which was in line with the induction of iNKT cell anergy observed in mouse studies. The anergy of iNKT cells in these cases may have been related to the fact that also non-professional APCs presented α-GalCer to iNKT cells (16). An attractive alternative route of administration to overcome these problems might be the skin. Here, α-GalCer would be taken up predominantly by skin-residing DCs or DCs in skin-draining lymph nodes. A study performed by Bontkes et al. compared the effect of intradermal versus intravenous injections and indeed showed prevention of iNKT cell anergy by intradermal injection of α-GalCer (17). Furthermore, intradermal α-GalCer triggered an earlier iNKT cell response and an increase in systemic iNKT cell numbers, leading to enhanced protective immunity in response to intradermal vaccination with protection against tumor outgrowth in five out of six mice. To add to this, Tripp et al. showed presentation of α-GalCer directly to iNKT cells in the draining lymph nodes in an *in vivo* mouse model, thereby bypassing migratory DCs and possibly iNKT cell anergy (18). Intranasal injection of α-GalCer was also shown to effectively reduce iNKT cell anergy in an *in vivo* mouse model as repeated dosing of α-GalCer *via* this route boosted iNKT cells and DCs without inducing anergy, as opposed to the intravenous route (19).

## Analogs of α-GalCer

As a result of the limited effects of α-GalCer in clinical studies, subsequent research focused on the development of glycolipid analogs with more distinct iNKT cell activating properties. Whereas some of these glycolipids predominantly induce Th2 type cytokine production in iNKT cells and were suggested to be mainly of potential use in auto-immune diseases (e.g., OCH and α-GalCer20:2), other glycolipid activators (e.g., those encompassing an aromatic ring in either the acyl- or shingosine tail) induced a predominant Th1 type immune response (20). Such Th1-biased glycolipids are more effective in triggering TCR activation and iNKT cell expansion compared to α-GalCer (21). These Th1 biased analogs include, e.g., α-C-GalCer and 7DW8-5. α-C-GalCer is a C-glycoside analog of α-GalCer and harbors a methyl group instead of a glycosidic oxygen. In a mouse melanoma metastasis model, α-C-GalCer was found to increase IL-12 and IFN-γ production and to decrease IL-4 production in comparison with α-GalCer and in addition exerted a more potent prophylactic effect against lung metastasis (22). Also in combination with monoclonal antibodies targeting tumor necrosis factor-related apoptosis-inducing ligand receptor (DR5) and 4-1BB, α-C-GalCer outperformed α-GalCer in experimental (established) mouse breast and renal tumors (23). Furthermore, while high concentrations of α-GalCer led to toxicity, this was not observed with α-C-GalCer.

The synthetic α-GalCer analog 7DW8-5 has a shorter fatty acid tail with a fluorinated benzene ring at the end and binds stronger to the CD1d molecule than α-GalCer (24). In vaccination studies, 7DW8-5 induced 100-fold stronger IFN-γ production by iNKT cells as compared with α-GalCer (24). When used as adjuvant for vaccination with tumor-associated antigens (TAAs) in a B cell lymphoma mouse model, IL-12 and IFN-γ production and an enhanced magnitude of the CD8^+^ T cell response were observed leading to an enhanced antitumor response (25).

## Glycolipid-Loaded APCs

Professional APCs are well equipped to provide optimal stimulatory signals to T cells that recognize their cognate antigen and are thereby capable of mediating antigen-specific immune responses against various targets. This potential of APCs could be used as a means to further strengthen the antitumor effect of glycolipids. Indeed, APCs that were loaded with α-GalCer *ex vivo* enhanced antitumor immune responses compared to α-GalCer alone in a B16 melanoma mouse model (26). This observation triggered multiple clinical phase I studies using mature or immature DCs pulsed with α-GalCer. Nieda et al. started the first phase I clinical trial where they administered α-GalCer pulsed immature moDCs to 12 patients with metastatic malignancies (27). They found increased serum IL-12 and IFN-γ levels and activated T and NK cells, indicating that NKT cells indeed bridged innate and adaptive immunity. This group performed another phase I clinical trial involving 12 patients with metastatic solid tumors (28). Effective iNKT cell activation was observed using immature moDCs pulsed with α-GalCer. Therapy was well tolerated and the majority of the patients experienced disease stabilization. Of note, intravenously administered α-GalCer pulsed DCs induced greater immunological effects compared to intradermally administered α-GalCer pulsed DCs. Several phase I and II clinical trials have been performed focusing on lung cancer using either α-GalCer pulsed DCs, peripheral blood mononuclear cells (PBMCs), or APCs (29–31). No adverse effects were observed and treatment was found to be safe and well tolerated. Chang et al. performed a phase I study in advanced cancer patients using intravenous administration of mature α-GalCer pulsed moDCs (32). Activation and a persistent expansion of the iNKT cell pool in combination with signs of secondary activation of other immune cell populations (including B cells, NK cells, and T cells) were observed as well as an increase in serum levels of IL-12 and IFN-γ. Interestingly, in a phase I clinical trial with asymptomatic myeloma patients, combination of low-dose lenalidomide with α-GalCer pulsed mature moDCs led to increased activation of innate immune cell subsets including iNKT, NK cells, monocytes, and eosinophils and a reduction in tumor-associated monoclonal immunoglobulin in three of four patients with measurable disease (33). Gasser et al. administered autologous moDCs loaded with α-GalCer, synthetic long peptides spanning immunogenic regions of the cancer-testis antigen NY-ESO-1, and short MHC-I-binding peptide sequences from the influenza virus intravenously in eighth high-risk stage II–IV melanoma patients (34). In three of these patients, a significant increase of peripheral iNKT cells was observed and four patients showed increased frequencies of IFN-γ positive cells when PBMCs were re-stimulated with α-GalCer. Five melanoma patients showed increases in cytokines related to α-GalCer stimulation found in the serum, and an increase in circulating NY-ESO-1-specific T cells was detected in seven patients.

To further enhance the effect of α-GalCer loaded APCs, combinations with chemotherapeutic agents known to induce immunogenic cell death were investigated. These chemotherapeutics can promote immune responses against the tumor by inducing activation of multiple cell death pathways and by enhancing the subsequent uptake of tumor peptides by APCs in the context of damage-associated molecular patterns (35). For example, gemcitabine and mafosfamide were tested in combination with α-GalCer-loaded bone marrow-derived DCs in a murine metastatic breast cancer model (36). Chemotherapy alone resulted in an increase in tumor cell CD1d expression, facilitating recognition by iNKT cells. Furthermore, α-GalCer-loaded DCs in combination with gemcitabine or mafosfamide led to increased IFN-γ production and a significant increase in survival.

## Incorporation of Glycolipids in Nanovectors

Another approach that is being explored with the aim to enhance the effect of α-GalCer entails its incorporation into nanovectors, which can act as vaccine carriers to induce an immune response by delivering their content to endosomes. Presentation of α-GalCer by CD1d-expressing APCs was indeed improved using liposomes and resulted in increased expansion and IFN-γ production by iNKT cells and a potent anti-metastatic effect in a highly malignant metastatic lung murine cancer model (37). Khan et al. used liposomes incorporating glycosphingolipids isolated from *Spingomonas paucimobilis*, which can, like α-GalCer, specifically activate iNKT cells (38). When these liposomes were loaded *ex vivo* onto bone marrow-derived DCs and used as treatment for mice with dimethyl-α-benzanthracene-induced tumors, a more sustained secretion of IFN-γ and a potent antitumor response was induced compared to administration of glycosphingolipids alone. A different approach consists of iNKT cell activation *via* targeted delivery of α-GalCer and OVA or tumor self-antigens (PLGA)-based nanoparticles that target the endocytic pathway of the cross-presenting CD8α^+^ DC subset *via* DEC205 (39) or Clec9a (40). Delivery of α-GalCer to CD8α^+^ DCs *via* this route enhanced iNKT cell transactivation of NK and T cells and a cytotoxic T cell response in *in vivo* mouse models and could promote both prophylactic and therapeutic antitumor responses in an advanced solid tumor model in mice. Notably, this approach could also target human CLEC9A-expressing DC to mediate the expansion of tumor self-antigen specific CD8^+^ T cells in PBMCs samples of melanoma patients *in vitro*, thereby underscoring the translational potential of this approach.

## CD1d-Inducing Agents

As iNKT cells can directly kill CD1d-expressing tumor cells, one can hypothesize that the efficacy of iNKT cell-based antitumor responses can also be improved by increasing CD1d-expression levels on tumor cells as this may facilitate their recognition by iNKT cells. It has been reported that inhibitors of histone deacetylases that regulate expression, cell cycle progression, and cellular proliferation are able to induce CD1d expression levels (41). Next to this, all-trans retinoic acid and certain chemotherapeutics have also been reported to increase CD1d expression levels and are, therefore, of potential interest either alone or in combination with other iNKT cell-based therapeutic approaches (36, 42).

## Adoptive Transfer of iNKT Cells

It is known that relatively low numbers of iNKT cells are present in peripheral blood of healthy individuals. This number is further reduced in many, but not all, cancer types (43–46). A higher number of circulating iNKT cells predicted improved outcome in head and neck squamous cell carcinoma (HNSCC) patients treated with curative-intent radiotherapy (47). Several studies were designed to increase the size of the iNKT cell population. Mouse *in vivo* studies support this strategy as adoptive transfer of murine iNKT cells, activated with IL-12 *ex vivo*, showed a potent antitumor response in a B16 melanoma and a lung metastasis model (48). Adoptive transfer of iNKT cells has the added advantage of reversing the defective iNKT cell IFN-γ production commonly observed in cancer patients, which is known to be important for promoting antitumor immune responses (45, 49, 50).

Several clinical trials have been performed using adoptive transfer of iNKT cells. In a phase I study of patients with advanced melanoma, Vα24 iNKT cells were isolated from patients PBMCs and *ex vivo* expanded for several weeks (45). After adoptive transfer, an increased number of iNKT cells and an increased activation state of iNKT cells and other immune cell subsets was observed without signs of toxicity. A slightly different approach was used in clinical trials in patients with HNSCC. Here, iNKT cells were isolated from PBMC and expanded *ex vivo* with α-GalCer and IL-2, while APC fractions were generated from PBMC by culturing them in the presence of GM-CSF and IL-2 (51). Expanded iNKT cells were then intra-arterially infused in the tumor-feeding artery while α-GalCer pulsed APCs were injected in the nasal submucosa. Therapy was found to be safe and resulted in an objective response rate of 50%. Increased intra-tumoral accumulation of transferred iNKT cells was associated with improved clinical outcome. Additional clinical trials were designed combining administration of expanded iNKT cells with α-GalCer pulsed DCs in patients with recurrent HNSCC reviewed by Motohashi et al. (52). Again, combination therapy appeared to exert beneficial clinical effects with disease stabilization and tumor regression associated with increased intratumoral iNKT cell numbers (53, 54). Based on these positive results, additional trials involving the adoptive transfer of iNKT cells in various tumor types were initiated, the results of which are eagerly awaited (NCT03093688; NCT02619058; NCT01801852).

## Tumor Targeting of iNKT Cells

All strategies described above are based on either the intravenous or intra-arterial administration of iNKT cells or the systemic or intradermal/intranasal activation of iNKT cells. Although these approaches can trigger antitumor immune responses, antitumor activity may be more pronounced and consistent when one can specifically target and activate iNKT cells in the tumor microenvironment. The potential of this approach has been demonstrated using a bispecific molecule generated by genetic fusion of a single chain variable fragment (scFv) targeted to a specific tumor peptide and CD1d, which can be loaded with specific glycolipids to allow iNKT cell activation. The antitumor activity of such a bispecific approach outperformed the activity of α-GalCer as was demonstrated *in vivo* in mice inoculated with Her2 or CEA expressing tumors using Her2- and CEA-targeted constructs, respectively (55–57). iNKT, NK, and T cells were found to accumulate at the tumor site using these targeted approaches and, in addition, treatment was not accompanied by iNKT cell anergy as iNKT cells remained responsive to repeated injections of the CD1d fusion proteins loaded with α-GalCer (55, 56).

## Chimeric Antigen Receptor (CAR) iNKT Cells

Another strategy combining tumor targeting of iNKT cells with an increase in the size of the iNKT cell population consists of adoptive transfer of CAR-expressing iNKT cells. CAR therapies were first applied to conventional T cells resulting in the approval by the Food and Drug Administration of two CAR-T cell therapies for hematological malignancies: one for acute lymphoblastic leukemia and one for advanced lymphoma. The currently used CARs consist of a scFv for antigen binding, the TCR ζ chain for TCR activation, and one or two signaling domains from the co-stimulatory molecules CD28 and/or 4-1BB (58). After the introduction of the CAR, there is still a large diversity in TCR specificity and function among conventional CAR-T cells, whereas CAR iNKT cells (due to the invariant nature of their TCR) constitute a more homogenous population with respect to both their function and specificity to CD1d, and this may translate into a different and perhaps more predictable and manageable toxicity profile (58).

Invariant natural killer T cells were reported by Heczey et al. to be a safe and effective platform for CAR redirected cancer immunotherapy in neuroblastoma (58). This approach showed effective *in vitro* cytotoxicity of Vα24 human iNKT cells with a CAR targeting the ganglioside GD2 antigen expressed by neuroblastoma cells. Also, iNKT cells retained their ability to kill tumor-associated macrophages as a result of TCR-mediated recognition of CD1d. Using this therapeutic approach in a neuroblastoma mouse model, transfer of GD2-specific CAR iNKT was shown to induce antitumor activity resulting in prolonged survival of mice. Of importance, GD2-specific CAR iNKT cells did not lead to graft versus host disease even after repeated infusions.

In a B cell lymphoma model, Tian et al. demonstrated that CD19-specific CAR-iNKT cells expressing CD62L, a ligand involved in homing of naïve and central memory T cells to secondary lymphoid organs, was the predominant CAR iNKT population that mediated tumor regression (59). This potentially allows for future selection of a more effective CAR-iNKT approach. A phase I clinical trial is currently ongoing wherein children with neuroblastoma are treated with GD2 CAR and IL-15 expressing autologous iNKT cells (NCT03294954).

## CD1d-Specific Antibodies

Instead of targeting iNKT cells that interact with CD1d, effects of monoclonal antibodies specific for CD1d have also been explored. Yue et al. showed that direct ligation of CD1d by monoclonal antibodies on the DC could, at least in part, mimic iNKT cell help to DCs (60). Ligation of several monoclonal CD1d antibodies led to downstream signaling *via* NF-κB, resulting in IL-12 production and moDC maturation. Recently, CD1d-specific single domain antibodies (sdAb) have been identified with a similar ability to induce DC maturation and IL-12 production *via* CD1d ligation (61). sdAb have several advantages over conventional monoclonal antibodies, including extended stability, low immunogenicity, ease of production and, due to their small size (15 kDa), deep and homogenous tumor penetration (62). As sdAbs can also be easily cloned to other molecules, e.g., TAAs, this can provide a vaccine encompassing a stimulatory signal to DCs, which will promote the efficient initiation and development of a tumor-associated antigen specific cytotoxic T cell response.

## Concluding Remarks

**Figure 1:**
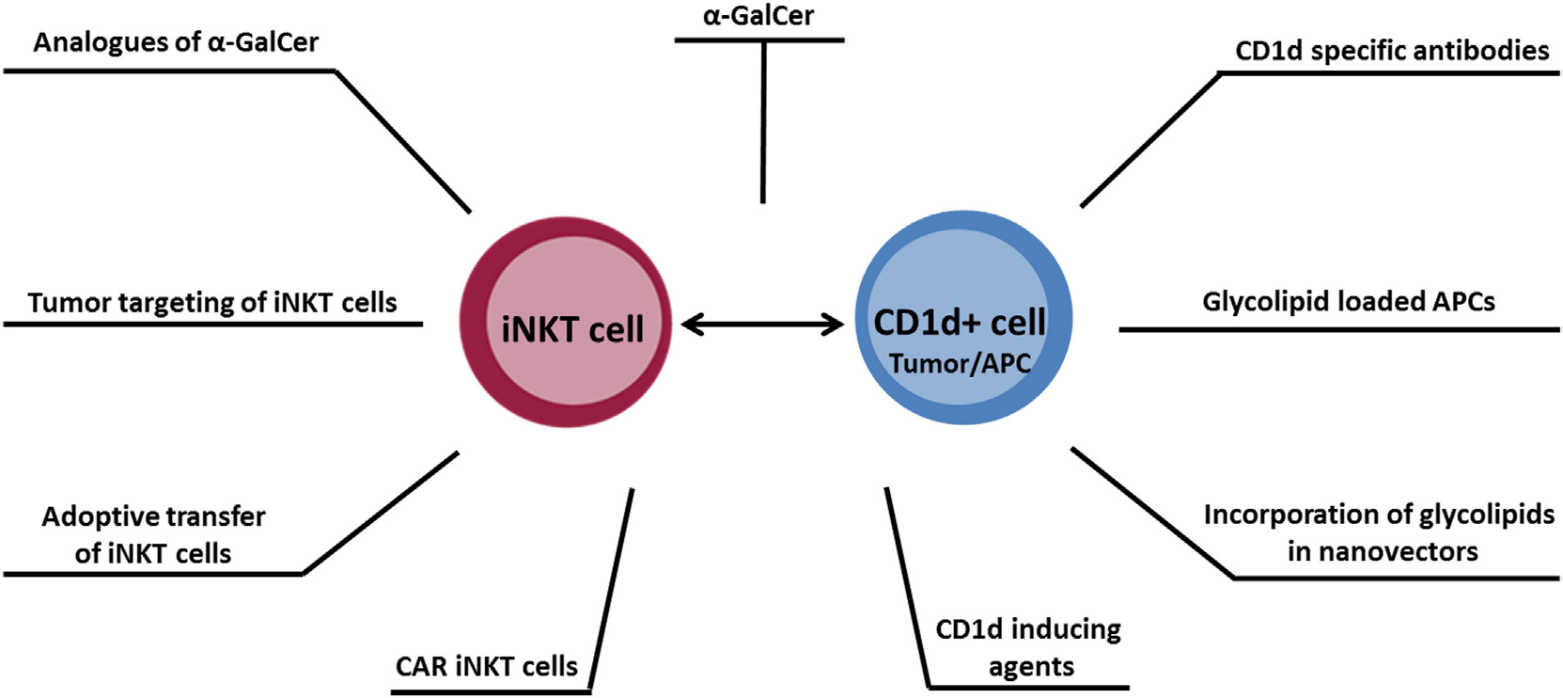
Overview of antitumor therapeutic approaches designed to target the CD1d–invariant natural killer T cell axis.

Within the last two decades, the role of iNKT cells within the antitumor immune response has been intensely studied. It is now recognized that iNKT cells play an important role in orchestrating immune responses, making strategies exploiting these cells potentially valuable for cancer immunotherapy. Several approaches for therapeutic manipulation of iNKT cells are being explored (illustrated in Figure 1), which may ultimately translate into a more effective therapy for cancer.

## Author Contributions

LK wrote the manuscript. HV co-wrote and reviewed the manuscript. RL and TG reviewed the manuscript.

## Conflict of Interest Statement

LK and Rl are funded by Lava Therapeutics. HV acts as chief scientific officer of Lava Therapeutics. The remaining author declares that the research was conducted in the absence of any commercial or financial relationships that could be construed as a potential conflict of interest.
