# Corrigendum: CD1d-Invariant Natural Killer T Cell-Based Cancer Immunotherapy: α-Galactosylceramide and Beyond

**DOI:** 10.3389/fimmu.2018.02606

**Published:** 2018-11-20

**Authors:** Lisa A. King, Roeland Lameris, Tanja D. de Gruijl, Hans J. van der Vliet

**Affiliations:** ^1^Department of Medical Oncology, VU University Medical Center and Cancer Center Amsterdam, Amsterdam, Netherlands; ^2^Lava Therapeutics, Den Bosch, Netherlands

**Keywords:** iNKT cells, CD1d, α-GalCer, glycolipids, cancer immunotherapy

In the original article, we neglected to disclose that authors Lisa A. King and Roeland Lameris are currently funded by Lava Therapeutics and that Hans J. van der Vliet also acts as chief scientific officer of Lava Therapeutics. Hans J. van der Vliet's affiliation has been updated to reflect this. The corrected Conflict of Interest statement appears below.

## Conflict of interest statement

LK and Rl are funded by Lava Therapeutics. HV acts as chief scientific officer of Lava Therapeutics. The remaining author declares that the research was conducted in the absence of any commercial or financial relationships that could be construed as a potential conflict of interest.

The authors apologize for this error and state that this does not change the scientific conclusions of the article in any way. The original article has been updated.

### Conflict of interest statement

LK and Rl are funded by Lava Therapeutics. HV acts as chief scientific officer of Lava Therapeutics. The remaining author declares that the research was conducted in the absence of any commercial or financial relationships that could be construed as a potential conflict of interest.

